# Anatomical Hepatectomy in a Patient with Situs Inversus Totalis and Right-Sided Round Ligament

**DOI:** 10.70352/scrj.cr.25-0361

**Published:** 2025-10-29

**Authors:** Makoto Shinzeki, Yu Asakura, Kaori Tokuhara, Masaharu Fukushima, Kento Ueda, Koji Ueta, Susumu Miura, Koichi Murata, Takeo Nomi

**Affiliations:** Department of Gastroenterological Surgery, Osaka Saiseikai Nakatsu Hospital, Osaka, Osaka, Japan

**Keywords:** situs inversus, right-sided round ligament, right-sided ligamentum teres, hepatectomy

## Abstract

**INTRODUCTION:**

Situs inversus totalis (SIT) is a rare congenital anomaly characterized by mirror-image reversal of the entire thoracoabdominal viscera. Right-sided round ligament (RSRL) is another rare congenital anomaly often associated with intrahepatic vascular variations. The coexistence of these 2 anomalies is extremely rare and presents a significant challenge for hepatectomy. Here, we report a case of anatomical hepatectomy performed in a patient with SIT and RSRL.

**CASE PRESENTATION:**

A 50-year-old Japanese man was diagnosed with ascending colon cancer and multiple lung and liver metastases. CT revealed SIT and RSRL. The patient underwent laparoscopic colectomy followed by chemotherapy with XELOX plus bevacizumab. We planned a hepatectomy for the residual liver metastasis after 4 courses of chemotherapy. To assess the intrahepatic vasculature accurately, we horizontally flipped the CT and 3D images to correct the mirror-reversed orientation of the liver, creating standard anatomical images without SIT. We then standardized the anatomical terminology within the surgical team to prevent misinterpretation of structures during surgery. We performed anatomical hepatectomy of the dorsal area of the right paramedian sector to resect residual hepatic lesions after chemotherapy. The procedure was performed on the right side of the patient using a standard surgical approach. Histopathological examination identified a single viable nodule with hepatic metastasis, whereas the remaining nodules showed a pathological complete response. The patient recovered uneventfully and was discharged on POD 10. The patient remained alive without disease progression 84 months after hepatectomy.

**CONCLUSIONS:**

We successfully performed anatomical hepatectomy in a patient with SIT and RSRL. Normalized imaging and standardization of anatomical terminology within the surgical team are key to ensuring surgical precision, preventing confusion during the operation, and avoiding potentially fatal complications of SIT and RSRL.

## Abbreviations

P_RPM_
: right paramedian portal pedicle
RSRL
: right-sided round ligament
SIT
: situs inversus totalis
V8d
: drainage vein of the dorsal part of segment VIII
V8i
: drainage vein running on the intermediate plane between the ventral and the dorsal part of segment VIII

## INTRODUCTION

SIT is a rare congenital anomaly in which the thoracoabdominal viscera are in a mirror-reversed position. The reported frequency of SIT ranges from 1:5000 to 1:10000.^1)^ RSRL is another rare congenital anomaly often associated with intrahepatic vascular variations.^2–5)^ The coexistence of these 2 anomalies is extremely rare and poses significant surgical challenges. Here, we report a case of anatomical hepatectomy performed in a patient with SIT and RSRL.

## CASE PRESENTATION

**Fig. 1:**
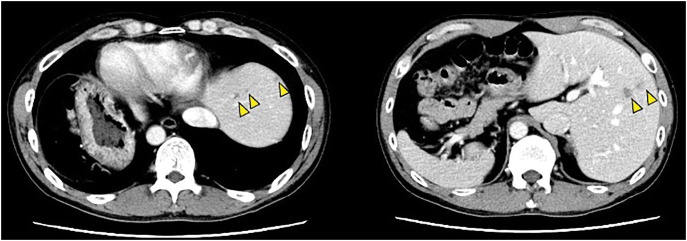
Contrast-enhanced CT showing multiple liver metastases (triangles).

**Fig. 2:**
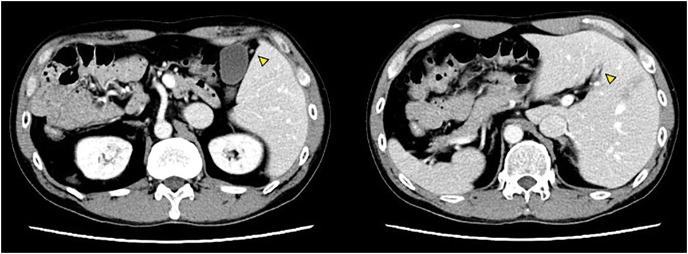
The round ligament (triangle) is connected to the right anterior portal vein through the outer side of the gallbladder.

A 50-year-old man presented at our hospital with abdominal distension and nausea. Colonoscopy revealed ascending colon stenosis caused by advanced colon cancer. Contrast-enhanced CT showed multiple lung and liver metastases (**Fig. 1**). CT revealed that all the viscera and vessels were in a mirror-reversed position. Additionally, the round ligament containing the fetal umbilical vein was connected to the right anterior portal vein, with the gallbladder positioned medially to the round ligament (**Fig. 2**). The right lateral portal pedicle branched directly from the main portal vein. These findings indicated the coexistence of SIT and RSRL.

The patient underwent chemotherapy with the XELOX regimen (capecitabine plus oxaliplatin) combined with bevacizumab after laparoscopic colectomy. After 4 courses of chemotherapy, all lung metastases shrunk, and most of the liver metastases disappeared. We planned hepatectomy for the residual liver metastasis.

**Fig. 3:**
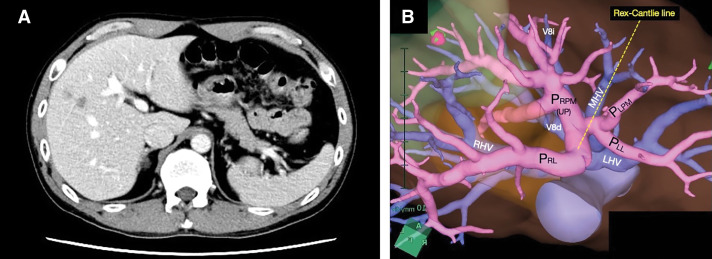
Horizontally flipped CT images correct the mirror-reversed position of the liver, creating standard anatomical images without SIT (**A**). Adjusted 3D images using a simulation imaging system provide precise information about the intrahepatic vasculature (**B**). LHV, left hepatic vein; MHV, middle hepatic vein; P_LL_, left lateral portal pedicle; P_LPM_, left paramedian portal pedicle; P_RL_, right lateral portal pedicle; P_RPM_, right paramedian portal pedicle; RHV, right hepatic vein; V8d, drainage vein of the dorsal part of segment VIII; V8i, drainage vein running on the intermediate plane between the ventral and the dorsal part of segment VIII

We conducted a detailed preoperative examination of the anatomical structures of the viscera and vessels. To enhance spatial recognition, we horizontally flipped the CT images to correct the mirror-reversed position of the liver, thereby creating standard anatomical images without the SIT. Similarly, we generated adjusted 3D images using a 3D simulation imaging system (SYNAPSE VINCENT; Fuji Film, Tokyo, Japan) to obtain precise information about the intrahepatic vasculature (**Fig. 3**). We planned a surgical strategy using the corrected images. In the corrected CT images, all residual liver metastases were located in the dorsal area of the right paramedian sector.

We standardized the anatomical terminology within the surgical team to prevent misinterpretation of the structures during surgery. During surgery, we designated mirror-reversed structures with the prefix “inverted” to ensure a shared understanding of anatomical structures within the surgical team. For example, the right hemiliver, which was actually located on the left side, was referred to as the “inverted” right hemiliver, and the right Glissonean pedicle, also situated on the left, was termed the “inverted” right Glissonean pedicle.

**Fig. 4:**
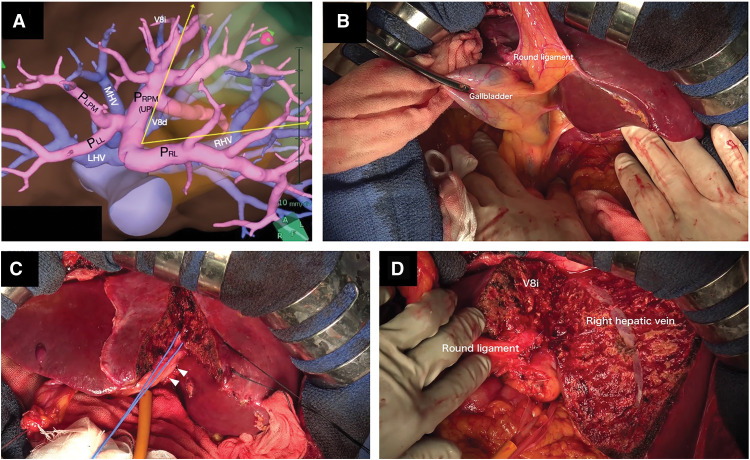
Anatomical hepatectomy of the dorsal region of the “inverted” right paramedian sector. Arrows indicate liver transection planes on 3D images (**A**). The gallbladder is situated medial to the round ligament (**B**). The dorsal branch of the right paramedian portal pedicle, connected to the round ligament (triangles), was taped (**C**). V8i and the right hepatic vein were preserved on the transected surface of the remnant liver (**D**). LHV, left hepatic vein; MHV, middle hepatic vein; P_LL_, left lateral portal pedicle; P_LPM_, left paramedian portal pedicle; P_RL_, right lateral portal pedicle; P_RPM_, right paramedian portal pedicle; RHV, right hepatic vein; V8d, drainage vein of the dorsal part of segment VIII; V8i, drainage vein running on the intermediate plane between the ventral and the dorsal part of segment VIII

During surgery, all the abdominal viscera and vessels were found to be in a reversed position. The surgeon performed hepatectomy on the patient’s right side. The patient underwent an anatomical hepatectomy of the dorsal area of the “inverted” right paramedian sector to remove the residual hepatic lesions (**Fig. 4**). During liver transection along the left side of the round ligament and “inverted” V8i, the dorsal branches of the “inverted” P_RPM_ and “inverted” V8d were divided. Furthermore, we transected the liver parenchyma along the “inverted” right hepatic vein, resecting some branches from the “inverted” right lateral portal pedicle. The operative time was 502 minutes, with an estimated blood loss of 1260 mL, and no transfusion was required during the perioperative period. The procedure was performed via laparotomy. Although a laparoscopic approach was considered, it was avoided because of the potential risk of misidentification of intrahepatic vessels due to the abnormal visceral positioning and the complex intrahepatic vascular anatomy associated with the coexistence of SIT and RSRL.

Histopathological examination revealed a single viable nodule of hepatic metastasis, whereas the remaining nodules showed a pathological complete response. The patient recovered uneventfully and was discharged on POD 10.

The patient underwent postoperative chemotherapy with XELOX plus bevacizumab and survived without disease progression for 84 months after hepatectomy.

## DISCUSSION

Situs inversus is a rare congenital anomaly characterized by the mirror-reversed positioning of the thoracoabdominal viscera and vessels. This anomaly is classified as SIT, in which the entire thoracoabdominal viscera are reversed, or partial situs inversus, in which only specific viscera are reversed. Katsuki et al.^6)^ reviewed 250 cases of situs inversus in Japan and reported an SIT-to-partial situs inversus ratio of 4.3:1. The reported frequency of SIT ranges from 1:5000 to 1:10000.^1)^ This clinical condition does not influence normal health or life expectancy.^7)^ However, it is often associated with congenital anomalies, including cardiovascular malformations, asplenia or polysplenia, hepatobiliary and pancreatic abnormalities, and intestinal malrotation.^8,9)^ Among these, cardiovascular anomalies are the most common, occurring in 73.1% of the patients with situs inversus.^6)^ The unusual positioning and altered spatial relationships of the viscera in situs inversus require surgeons to have a precise anatomical understanding and advanced technical skills.^9)^

RSRL is a relatively rare congenital anomaly, with a reported prevalence of 0.11%–1.2%, and often accompanied by intrahepatic vascular anomalies.^2–5)^ During embryonic development, the right umbilical vein normally regresses, whereas a remnant of the left umbilical vein remains, forming the umbilical portion of the portal system. However, in RSRL cases, the left umbilical vein disappears and the right umbilical vein develops, connecting to the right paramedian trunk of the portal vein. Consequently, the round ligament deviates to the right, and the gallbladder is positioned to the left of the round ligament.^10,11)^ This anomaly has been recognized as a displacement of the gallbladder and is termed left-sided gallbladder.^2,12)^ Subsequent reports suggest that the cause of RSRL is not malposition of the gallbladder, but rather the development of the residual right portal vein.^2,5)^ Shindoh et al. reported that the gallbladder is located along the sectoral border between the right and left hemilivers, even in RSRL.^2)^

Coexistence of SIT and RSRL is extremely rare. Ibukuro et al. analyzed 14384 patients who underwent contrast-enhanced CT for various conditions in detail.^3)^ Their study identified 6 cases of situs inversus, 19 cases of RSRL, and 3 cases of both conditions. RSRL, also known as left-sided gallbladder, was initially defined as the displacement of the gallbladder to the left of the round ligament without situs inversus.^2,13,14)^ Ibukuro et al. proposed the term “corrected RSRL” for RSRL with situs inversus, as identified on flipped images, to distinguish it from cases where the round ligament and gallbladder are malpositioned owing to RSRL alone.^3)^ In our case, we confirmed RSRL using flipped CT images and diagnosed it with “corrected RSRL” in the presence of situs inversus.

Although sporadic clinical reports and embryological discussions of SIT and RSRL exist, their mechanisms remain unproven. SIT is thought to result from abnormal left–right axis determination during early embryogenesis, leading to a mirror-image reversal of visceral organ positioning. By contrast, RSRL is considered to result from anomalous regression of the left umbilical vein with persistence of the right umbilical vein, resulting in a round ligament that courses abnormally to the right hepatic lobe. The concurrence of these 2 rare anomalies may reflect overlapping disturbances in left–right patterning and umbilical vein development. Specifically, the reversal of visceral arrangement in SIT could potentially influence the remodeling process of the umbilical veins, predisposing to regression of the “inverted” left umbilical vein and persistence of the contralateral vessel. However, no definitive embryological pathway has been established, and further investigation is required to clarify whether their coexistence represents a coincidental association or a common developmental mechanism.

To our knowledge, this is the first reported case of hepatectomy in a patient with SIT and RSRL. The coexistence of SIT and RSRL is associated with significant surgical difficulties. The unusual positioning and altered spatial relationships of the viscera in SIT can lead to disorientation and misinterpretation, thereby increasing the risk of serious complications. Additionally, in RSRL, the presence of anomalous vasculature may further complicate intraoperative navigation, thereby increasing the likelihood of surgical difficulties.

We implemented 2 preoperative steps to ensure surgical safety. The 1st step was a careful preoperative anatomical assessment. Although SIT is easily recognizable on CT, RSRL can be more challenging to diagnose. This difficulty arises because in RSRL, the gallbladder does not always appear on the left side of the round ligament, making diagnosis more difficult. A previous study reported that among 18 patients with RSRL, the gallbladder was located to the right (27.7%), below (38.8%), or left (33.3%) of the round ligament.^3)^ Some reports have described RSRL as being diagnosed intraoperatively.^15)^ Detecting RSRL in the presence of SIT is expected to be even more challenging because of its mirror-reversed anatomy, which can obscure its preoperative identification.

The CT images were flipped horizontally to correct the mirror-reversed orientation of the liver, thus creating standard anatomical images without the SIT. The 3D imaging simulation reconstructed from contrast-enhanced CT scans was also flipped horizontally to enhance the precise spatial recognition of the intrahepatic structures.

Next, we carefully standardized the anatomical terminology within the surgical team before surgery. Establishing consistent anatomical terms within the surgical team is crucial to prevent misinterpretation of structures, especially in situs inversus, where an altered orientation can lead to confusion during surgery.

These strategies enable safe hepatic resection without misidentifying the anatomy during surgery, thereby preventing injury to the preserved vessels and minimizing the risk of complications.

## CONCLUSIONS

We successfully performed an anatomical hepatectomy of the dorsal area of the “inverted” right paramedian sector in a patient with SIT and RSRL. Precise anatomical recognition using flipped CT and 3D images is crucial for accurate surgical planning. Standardizing the anatomical terminology within the surgical team helps prevent misdirection during surgery. Furthermore, when both anomalies coexist, meticulous preoperative planning and intraoperative caution are essential to avoid damage to the preserved structures and prevent potentially fatal complications.
